# LncRNA FBXL19-AS1 regulates the miR-650/ITGB3 axis to drive pathological progression in Alzheimer’s Disease

**DOI:** 10.1186/s41065-026-00727-1

**Published:** 2026-08-14

**Authors:** Jie Dou, Yanping Zhu, Zili Du, Guangning Zhang

**Affiliations:** 1 Rongjun Rehabilitation Department 5, Hebei Third Rongjun Youfu Hospital, Baoding, 071000 China; 2 Department of Neurology III, Taiyuan People’s Hospital, Taiyuan, 030025 China; 3 Neurosurgery Ward 2, Affiliated Hospital of Zunyi Medical University, No. 149, Dalian Road, Huichuan District, Zunyi, 563000 China; 4 Department of Neurosurgery, The First Hospital of Jiaxing, No. 1882, Zhonghuan South Road, Jiaxing, 314001 China

**Keywords:** Alzheimer's Disease, FBXL19-AS1, MiR-650, ITGB3, CeRNA, Neuroinflammation, Diagnostic biomarker

## Abstract

**Background:**

Alzheimer’s Disease (AD) is a progressive neurodegenerative disorder characterized by complex and not yet fully understood pathological mechanisms. This study was designed to explore the molecular mechanisms of long non-coding RNA (lncRNA) FBXL19-AS1 in the pathological progression of AD.

**Methods:**

110 AD patients and 110 healthy controls were recruited for this study. Relevant gene expression was measured using reverse transcription quantitative real-time PCR (RT-qPCR), and the diagnostic performance was assessed through receiver operating characteristic (ROC) curve analysis. AD cell models were established by treating SH-SY5Y and BV2 cells with amyloid β (Aβ)25–35. Cell proliferation was evaluated using the cell counting kit-8 (CCK-8) assay, cell apoptosis was analyzed by flow cytometry, and the secretion levels of inflammatory factors were determined via enzyme-linked immunosorbent assay (ELISA). A dual-luciferase reporter assay was performed to confirm the direct regulatory interactions.

**Results:**

In AD patients, the FBXL19-AS1 and integrin subunit beta 3 (ITGB3) expression were significantly elevated, whereas microRNA-650 (miR-650) expression was markedly reduced. FBXL19-AS1 showed certain diagnostic potential for AD, and its expression was negatively correlated with Montreal Cognitive Assessment (MoCA) scores. Following treatment with Aβ25–35, SH-SY5Y cells exhibited reduced proliferative capacity and increased apoptosis, while BV2 cells showed elevated levels of inflammatory factors. Additionally, Aβ25–35 treatment led to upregulated expression of FBXL19-AS1 and ITGB3, along with downregulated expression of miR-650. Knockdown of FBXL19-AS1 effectively reversed these effects. However, inhibition of miR-650 partially attenuated the changes induced by FBXL19-AS1 knockdown. Furthermore, knockdown of FBXL19-AS1 markedly reduced ITGB3 expression, whereas concurrent inhibition of miR-650 partially reversed this downregulatory effect.

**Conclusion:**

FBXL19-AS1 appears to interact with and modulate miR-650, thereby affecting ITGB3 expression and potentially participating in the pathological progression of AD.

**Supplementary Information:**

The online version contains supplementary material available at https://doi.org/10.1186/s41065-026-00727-1.

## Background

Alzheimer’s Disease (AD) is a severe neurodegenerative disorder [1], primarily characterized by cognitive impairment and memory decline [2, 3]. Its pathological hallmarks include brain tissue degeneration, such as neuronal loss, abnormal protein accumulation (β-amyloid), and the formation of neurofibrillary tangles [4, 5]. The pathogenesis of AD is complex and multifactorial, involving genetic, environmental, and metabolic influences, and is associated with a highly heterogeneous pathological progression [6, 7]. As the global aging population continues to grow, the social and economic burdens of AD are expected to rise significantly, placing substantial psychological and financial strain on patients’ families and society at large [8, 9]. However, reliable early diagnostic biomarkers and effective disease-modifying therapies remain lacking.

Long non-coding RNAs (lncRNAs) [10] can function as competitive endogenous RNAs (ceRNAs) by sponging microRNAs (miRNAs), thereby modulating the expression of target genes [11, 12]. Non-coding RNAs play crucial roles in AD by influencing key pathological mechanisms [13–16], including Aβ metabolism, tau protein phosphorylation, and neuroinflammation [14]. For instance, silencing of XIST attenuates the expression of the AD-related BACE1 via miR-124 [17], whereas BDNF-AS promotes BACE1 expression by competitively binding to miR-9-5p, thereby exacerbating amyloid deposition [18]. Recent RNA sequencing and bioinformatics analyses have identified FBXL19-AS1 as one of the differentially expressed lncRNAs in AD [19]. However, its specific regulatory mechanism in the context of AD remains uncharacterized. Studies have demonstrated that miR-650 is downregulated in AD, and its overexpression can alleviate AD-related pathological changes [20]. Moreover, miR-650 levels are associated with apoE concentration, a major risk factor for AD [21]. Meanwhile, integrin subunit beta 3 (ITGB3) is upregulated in astrocytes of AD mouse models and is closely linked to neuroinflammatory processes [22]. Bioinformatics analyses predict that FBXL19-AS1 could directly target miR-650, which in turn targets ITGB3. Therefore, we hypothesized that FBXL19-AS1 may regulate ITGB3 expression through miR-650, thereby contributing to the pathogenesis of AD.

Through clinical sample analysis and in vitro cell experiments, this study systematically investigated the regulatory mechanisms and functional roles of the FBXL19-AS1/miR-650/ITGB3 axis in AD, offering novel theoretical insights for early diagnosis and therapeutic strategies in AD.

## Methods

### Participants

110 individuals diagnosed with AD at Affiliated Hospital of Zunyi Medical University, based on the National Institute on Aging and Alzheimer’s Association (NIA-AA) diagnostic criteria [23], were recruited for this study. Additionally, 110 healthy controls matched for age and gender were included. All participants had at least 12 years of formal education and were free from other neurological disorders, severe cardiovascular or cerebrovascular diseases, malignant tumors, or any other factors that could potentially interfere with cognitive performance.

The Montreal Cognitive Assessment (MoCA) was administered to evaluate the cognitive function of the participants. In addition, peripheral venous blood samples were collected from all participants. Blood was drawn into vacuum blood collection tubes containing clot activator (BD Vacutainer, Becton, Dickinson and Company, Franklin Lakes, NJ, USA). After collection, the tubes were allowed to stand at room temperature for 30 min to ensure complete clot formation. The blood was then centrifuged at 3000×g for 15 min at 4 °C. The separated serum was carefully aliquoted into sterile, nuclease-free microcentrifuge tubes and immediately stored at − 80 °C until further analysis. 

This study was approved by the Ethics Committee of Affiliated Hospital of Zunyi Medical University (Approval Number: KLL-2022-758) and performed in line with the principles of the Declaration of Helsinki, and all participants provided written informed consent.

### Cell culture and transfection

Human neuroblastoma SH-SY5Y cells and microglial BV2 cells were obtained from American Type Culture Collection (ATCC, Manassas, VA, USA) and cultured in Dulbecco’s Modified Eagle Medium/Nutrient Mixture F-12 (DMEM-F12, Gibco, Thermo Fisher Scientific, Waltham, MA, USA) medium supplemented with 10% fetal bovine serum (FBS, Gibco, Thermo Fisher Scientific). The cells were maintained at 37 °C in a humidified incubator containing 5% CO_2_.

To establish the in vitro models of neuronal injury and neuroinflammation, SH-SY5Y cells and BV2 microglia were treated with 40 µM Aβ25–35 (Sigma-Aldrich, St. Louis, MO, USA) for 24 h. This treatment regimen was selected according to previously reported protocols, which has been proven to successfully induce neuronal damage and microglial inflammatory responses in AD in vitro models [24].

si-FBXL19-AS1, miR-650 mimic, miR-650 inhibitor, and their respective negative controls were designed and synthesized by Shanghai GenePharma Co., Ltd (Shanghai, China). To test whether ITGB3 was the key downstream effector of the FBXL19‑AS1/miR‑650 axis, we co‑transfected an ITGB3 overexpression plasmid (OE‑ITGB3). Transfection was performed using Lipofectamine 2000 (Invitrogen, Thermo Fisher Scientific, Waltham, MA, USA) according to the experimental design, and the relevant parameters were assessed 48 h post-transfection. The final working concentration of the miR-650 inhibitor was 50 nM, as recommended by the manufacturer. To ensure experimental rigor and data reproducibility, all transfection experiments included a non-targeting negative control (si-NC or mimic-NC/inhibitor-NC). Additionally, a mock transfection group (without any oligonucleotides) was used to rule out any potential off-target effects. Each independent experiment was performed in triplicate, and the results were validated by at least three biological replicates.

### Reverse transcription quantitative real-time PCR (RT-qPCR)

Total RNA was extracted from peripheral blood or cultured cells using TRIzol reagent (Invitrogen, Thermo Fisher Scientific). RNA purity and concentration were assessed using a NanoDrop 2000 spectrophotometer (Thermo Fisher Scientific). The A260/A280 ratios of all samples ranged from 1.8 to 2.0, and the A260/A230 ratios were above 2.0, indicating no significant contamination by proteins, phenol, or salts. RNA integrity was further verified by the presence of sharp 18 S and 28 S ribosomal RNA bands on 1% agarose gel electrophoresis. Complementary DNA (cDNA) was synthesized via reverse transcription using the PrimeScript RT Reagent Kit (Takara Bio, Shiga, Japan). Using the synthesized cDNA as a template, RT-qPCR amplification was carried out using SYBR Green I Master Mix (Roche, Basel, Switzerland) on an Applied Biosystems 7500 Real-Time PCR System (Thermo Fisher Scientific). With glyceraldehyde-3-phosphate dehydrogenase (GAPDH) and U6 serving as internal reference genes, the relative expression of the target genes was calculated using the 2^⁻∆∆Ct^ method. All primer sequences for FBXL19-AS1, miR-650, ITGB3, GAPDH and U6 were designed and synthesized by GenePharma (Shanghai, China). The primer sequences were available from the corresponding author upon reasonable request. Each reaction was performed in triplicate.

### Proliferation assay

Transfected SH-SY5Y cells were seeded into 96-well plates at a density of 5 × 10^3^ cells per well. At 0, 24, 48, and 72 h post-seeding, 10 µL of cell counting kit-8 (CCK-8, Dojindo Laboratories, Kumamoto, Japan) reagent was added to each well. Following incubation at 37 °C for the appropriate time, the optical density (OD) value was measured at 450 nm.

### Apoptosis experiment

Treated SH-SY5Y cells were harvested, digested with trypsin, washed with phosphate-buffered saline (PBS), centrifuged, and resuspended. Subsequently, 10 µL of Annexin V-fluorescein isothiocyanate (FITC) /propidium iodide (PI) (BD Biosciences, Franklin Lakes, NJ, USA) staining solution was added, and the cells were incubated in the dark. Flow cytometric analysis was performed within 1 h using a BD FACSCalibur flow cytometer (BD Biosciences). For each sample, at least 10,000 events were acquired. The apoptosis rate was calculated using FlowJo software (v10.8, BD Biosciences). Cells that were Annexin V-FITC positive and PI negative (Annexin V⁺/PI⁻) were defined as early apoptotic cells, while cells that were double positive (Annexin V⁺/PI⁺) were defined as late apoptotic cells. The total apoptosis rate was calculated as the sum of the early apoptotic rate and the late apoptotic rate. All experiments were performed in triplicate.

### Enzyme-linked immunosorbent assay (ELISA)

The supernatant was collected from BV2 cells, and the levels of inflammatory factors- interleukin-6 (IL-6), interleukin-1β (IL-1β), and tumor necrosis factor-alpha (TNF-α)-were measured using ELISA kits (Invitrogen, Thermo Fisher Scientific, Waltham, MA, USA). The OD was read using a microplate reader, and the concentrations of the cytokines were determined based on the corresponding standard curves.

### Bioinformatics analysis of miR-650 target genes

To further explore the potential downstream mechanisms of miR-650 in AD, its target genes were predicted using ENCORI and TargetScan. The overlapping target genes from the two databases were subjected to Gene Ontology (GO) and Kyoto Encyclopedia of Genes and Genomes (KEGG) pathway enrichment analyses.

### Verification of the targeted relationship

Wild-type (WT) and mutant (MUT) pGL3 luciferase reporter plasmids (Promega, Madison, WI, USA) harboring the 3’ untranslated regions (3’UTRs) sequences of FBXL19-AS1 and ITGB3 were generated. These reporter constructs were co-transfected into cells alongside miR-650 mimics or inhibitors, and relative luciferase activity was quantified 48 h after transfection. To validate the specific binding interactions between miR-650 and FBXL19-AS1/ITGB3, the seed-binding motifs within predicted targeting sites were further mutated, with scrambled miRNA serving as negative controls. A Renilla luciferase reporter vector (pRL-TK, Promega) was co-transfected as an internal control for normalization. Forty-eight hours post-transfection, cells were lysed, and firefly luciferase and Renilla luciferase activities were measured sequentially using the Dual-Luciferase Reporter Assay System (Promega) on a GloMax 20/20 Luminometer (Promega). The relative luciferase activity was calculated as the ratio of firefly luciferase activity to Renilla luciferase activity for each well. All experiments were performed in triplicate.

### Western blot analysis

Total protein was extracted from SH-SY5Y and BV2 cells using RIPA lysis buffer (Beyotime, Shanghai, China) containing protease inhibitor cocktail (Roche, Basel, Switzerland). Protein concentration was determined using the BCA Protein Assay Kit (Thermo Fisher Scientific, Waltham, MA, USA). Equal amounts of protein (30 µg per lane) were separated by 10% SDS-polyacrylamide gel electrophoresis and transferred onto polyvinylidene difluoride (PVDF) membranes (Millipore, Billerica, MA, USA). The membranes were blocked with 5% non-fat milk in Tris-buffered saline containing 0.1% Tween-20 (TBST) for 1 h at room temperature, followed by overnight incubation at 4 °C with primary antibodies: anti-ITGB3 (1:1000, Abcam, Cambridge, UK) and anti-β-actin (1:5000, Abcam, Cambridge, UK) as a loading control. After washing with TBST, the membranes were incubated with HRP-conjugated secondary antibody for 1 h at room temperature. Protein bands were visualized using an enhanced chemiluminescence (ECL) substrate (Millipore) and quantified using ImageJ software (NIH, Bethesda, MD, USA).

### Subcellular fractionation and RNA distribution analysis

To determine the subcellular localization of FBXL19-AS1, nuclear and cytoplasmic fractions were separated using the PARIS™ Kit (Thermo Fisher Scientific, Waltham, MA, USA) according to the manufacturer’s instructions. Briefly, SH-SY5Y and BV2 cells (5 × 10⁶ cells) were harvested, washed with ice-cold PBS, and resuspended in cell fractionation buffer. After incubation on ice for 10 min, the lysates were centrifuged at 500×g for 5 min at 4 °C. The supernatant (cytoplasmic fraction) was carefully collected, and the nuclear pellet was washed once with fractionation buffer, then resuspended in nuclear lysis buffer. RNA was extracted from each fraction using TRIzol reagent (Invitrogen). The levels of FBXL19-AS1, U6 (nuclear control), and GAPDH mRNA (cytoplasmic control) were measured by RT-qPCR.

### Public database validation

To validate the expression changes of key genes in AD, we utilized the AlzData database (http://www.alzdata.org/), which integrates multiple GEO datasets (including GSE5281 and GSE48350) from human brain tissues (hippocampus and entorhinal cortex, Supplementary Fig. 5). The expression of ITGB3 was queried and compared between AD patients and healthy controls. For FBXL19‑AS1 and miR‑650, we referred to published studies that used independent public AD databases: Cai et al. (Gene, 2023) [19] validated the differential expression of FBXL19-AS1 in AD. (Mol Neurobiol, 2023) validated miR‑650 downregulation in AD [20]. These external data were used to support our clinical and cellular findings.

### Statistical analysis

Statistical analysis was conducted using SPSS 26.0 (IBM Corp., Armonk, NY, USA) in this study. Before applying parametric tests, the normality of continuous data was assessed using the Kolmogorov-Smirnov test. Homogeneity of variances was evaluated using Levene’s test. To compare differences between groups, the independent samples t-test was employed for normally distributed continuous variables, while the chi-square test was used for categorical variables. For comparisons involving more than two groups, one-way analysis of variance (ANOVA) followed by Tukey’s post hoc test was applied for normally distributed data, while the Kruskal-Wallis test with Dunn’s post hoc test was used for non-normally distributed data. The Pearson correlation coefficient was calculated to assess the linear relationship between continuous variables. The diagnostic performance was evaluated using receiver operating characteristic (ROC) curve analysis. A *P* < 0.05 was considered statistically significant. For all in vitro experiments, at least three independent biological replicates were performed (*n* = 3 per group), and data were presented as mean ± standard deviation (SD).

## Results

### Baseline clinical characteristics of the study participants

One hundred ten patients diagnosed with AD and 110 healthy controls were enrolled in the study. Comparative analysis of baseline clinical characteristics revealed no statistically significant differences between the two groups in terms of age, gender, body mass index (BMI), or smoking status. The mean MoCA score in the AD group was 17.38 ± 4.32 (Table 1).

**Table 1:** Clinical characteristics of the study population

Characteristics	Control group (*n* = 110)	AD group (*n* = 110)	*P* value
Age (years), mean ± SD	70.85 ± 4.01	71.25 ± 4.06	0.463
Gender, male/female	63/47 (57.3%/42.7%)	49/61 (44.5%/55.5%)	0.059
BMI (kg/m²), mean ± SD	22.90 ± 3.07	22.96 ± 2.97	0.876
Smoking, n (%)			0.257
Yes	21 (19.1%)	28 (25.5%)	
No	89 (80.9%)	82 (74.5%)	
MoCA score, mean ± SD	-	17.38 ± 4.32	

*AD* Alzheimer’s disease, *BMI* Body-mass index, *MoCA* Montreal cognitive assessment. P values were calculated using the independent samples t-test for continuous variables and the chi-square test for categorical variables

### Association between FBXL19-AS1 and AD

**Fig. 1:**
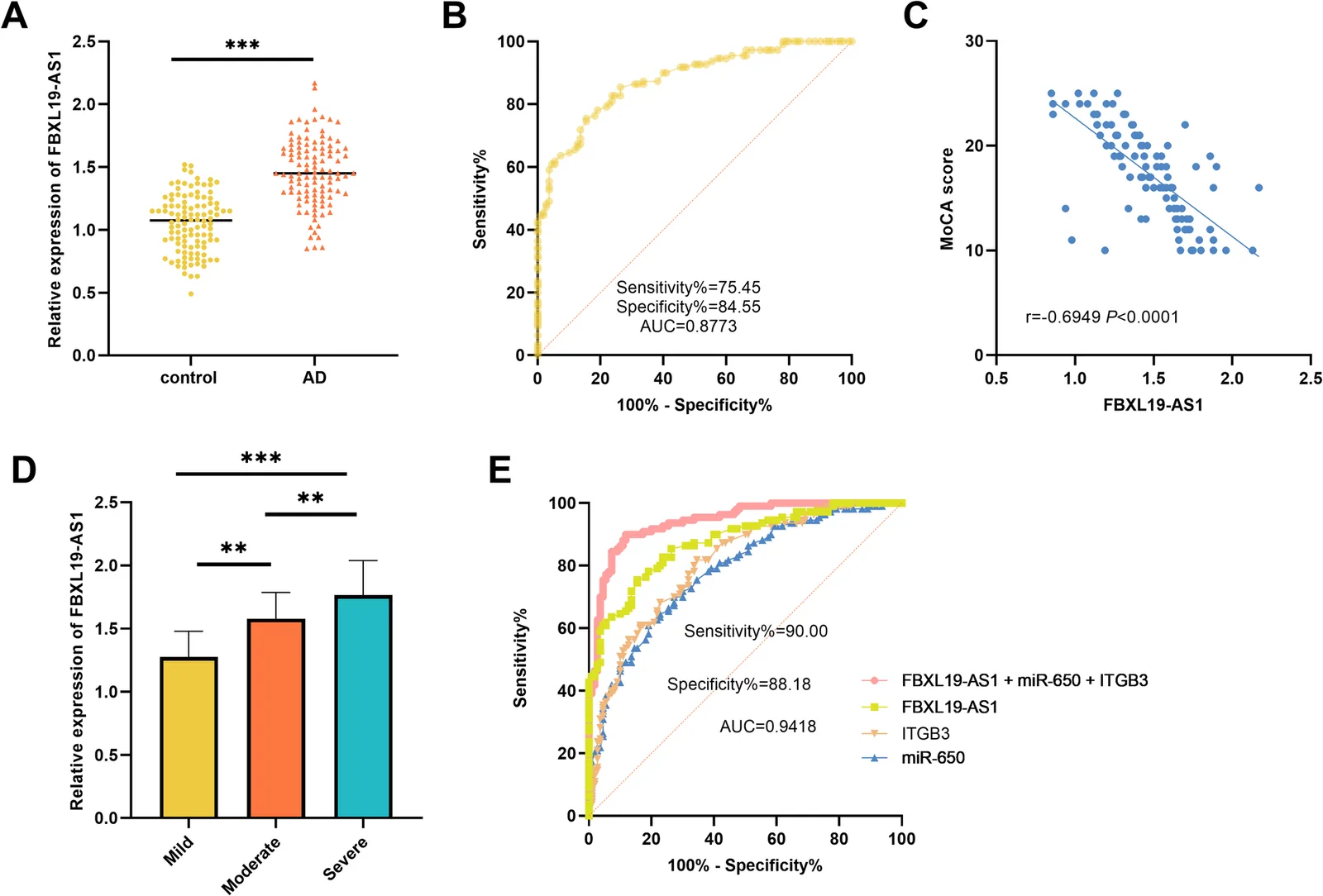
The association between FBXL19-AS1 and AD. **A**. High expression of FBXL19-AS1 may be associated with the onset of AD. **B**. FBXL19-AS1 exhibited potential diagnostic value for AD. **C**. FBXL19-AS1 expression correlated with cognitive function in AD patients. **D**. FBXL19-AS1 expression correlates with AD severity. **E**. ROC curve analysis evaluating the diagnostic performance of FBXL19-AS1, miR-650, ITGB3, and their combined model for AD. Statistical significance between groups in (A) was determined using the independent samples t-test. ROC curve analysis in (B) was performed to calculate AUC, sensitivity, and specificity. Pearson correlation analysis was used in (C). (D) was analyzed using one-way ANOVA followed by Tukey’s post hoc test for multiple group comparisons. All quantitative data are presented as mean ± standard deviation (SD). Each clinical sample was detected in three technical replicates. ** indicates *P* < 0.01. *** indicates *P* < 0.001

FBXL19-AS1 was highly expressed in AD (Fig. 1A) and exhibited promising diagnostic potential for AD, with a sensitivity of 75.45%, specificity of 84.55%, and an area under the curve (AUC) value of 0.8773 (Fig. 1B). The optimal cut-off value for FBXL19-AS1 expression was determined as 1.285 based on the Youden index, with an AUC 95% confidence interval (CI) of (0.8329–0.9217). Moreover, FBXL19-AS1 expression was significantly and negatively correlated with the MoCA scores of AD patients (*r* = -0.6949, *P* < 0.0001), suggesting its potential involvement in the progression of cognitive impairment in AD (Fig. 1C). Based on Montreal Cognitive Assessment (MoCA) scores, the 110 patients with AD were divided into three subgroups: mild AD (MoCA score: 19–25, *n* = 46), moderate AD (MoCA score: 11–18, *n* = 56), and severe AD (MoCA score ≤ 10, *n* = 8). We then compared the expression levels of FBXL19-AS1 across the three subgroups. As presented in Fig. 1D, the expression of FBXL19-AS1 increased progressively with the deterioration of AD severity (severe > moderate > mild), with statistically significant differences observed between every two groups (all *P* < 0.01). These findings indicated that FBXL19-AS1 expression was positively correlated with the clinical severity of AD. The ROC curves demonstrated that the combined diagnostic model of FBXL19-AS1, miR-650, and ITGB3 yielded an AUC of 0.9418 with a sensitivity of 90.00% and specificity of 88.18%, exhibiting superior diagnostic efficiency compared with each individual biomarker (Fig. 1E).

### FBXL19-AS1 in the Aβ25-35-induced AD cell model

**Fig. 2:**
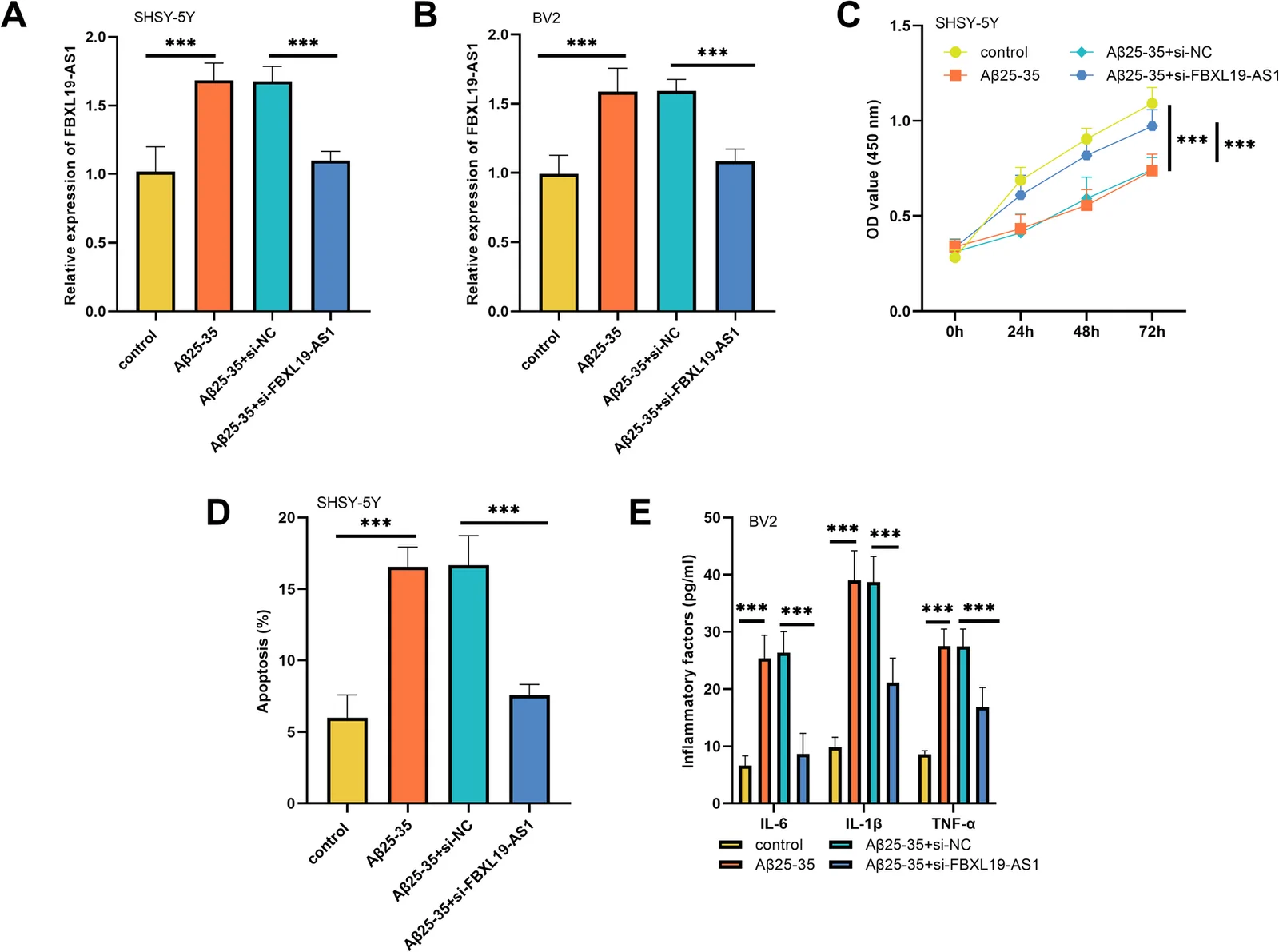
FBXL19-AS1 in the Aβ25-35-induced AD cell model. **A–B**. Aβ25–35 promoted FBXL19-AS1 expression in SH-SY5Y and BV2 cells, and si-FBXL19-AS1 effectively suppressed its expression. **C**. FBXL19-AS1 may enhance Aβ25–35-induced inhibition of neural cell proliferation, and knockdown of FBXL19-AS1 alleviates this effect. **D**. FBXL19-AS1 may contribute to the regulation of neural cell apoptosis induced by Aβ25–35. **E**. FBXL19-AS1 may promote microglial inflammatory responses induced by Aβ25–35, and its knockdown reduces inflammation. All data were analyzed using one-way ANOVA followed by Tukey’s post hoc test for multiple group comparisons. For all in vitro experiments, at least three independent biological replicates were performed (*n* = 3 per group), and data were presented as mean ± standard deviation (SD). *** indicates *P* < 0.001

Following Aβ25–35 treatment, FBXL19-AS1 expression was significantly upregulated, whereas transfection with si-FBXL19-AS1 effectively suppressed FBXL19-AS1 expression (Figs. 2A–B). Functional experimental results demonstrated that Aβ25–35 treatment markedly inhibited the proliferation capacity of SH-SY5Y cells (Fig. 2C), significantly increased the apoptosis rate (Fig. 2D), and promoted the release of inflammatory cytokines IL-6, IL-1β, and TNF-α from BV2 cells (Fig. 2E). Notably, knockdown of FBXL19-AS1 partially reversed these effects. To investigate whether FBXL19-AS1 localizes to the cytoplasm to function as a ceRNA, we performed subcellular fractionation followed by RT-qPCR in SH-SY5Y and BV2 cells. As shown in Supplementary Fig. 1, FBXL19-AS1 was predominantly localized in the cytoplasm.

### Targeting the relationship between FBXL19-AS1 and miR-650

**Fig. 3:**
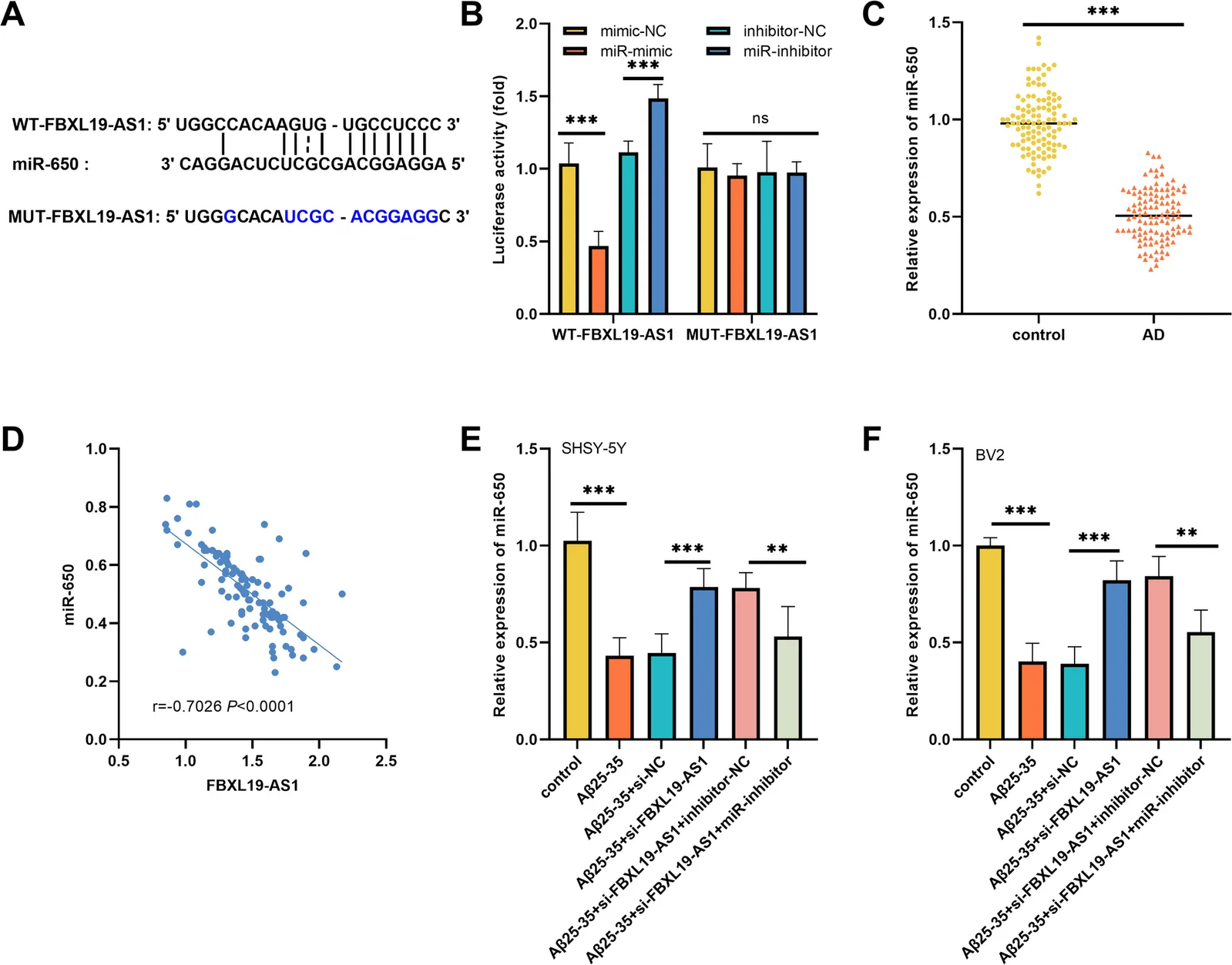
The targeting relationship between FBXL19-AS1 and miR-650. **A**. Complementary sequences between FBXL19-AS1 and miR-650. **B**. Luciferase reporter assays confirmed the direct interaction between FBXL19-AS1 and miR-650. **C**. miR-650 expression may be downregulated during AD pathogenesis. **D**. FBXL19-AS1 and miR-650 expression levels were inversely correlated. **E–F**. In Aβ25–35-treated SH-SY5Y and BV2 cells, miR-650 expression was measured following FBXL19-AS1 intervention. For items (B, E, F), a single-factor analysis of variance (with Tukey’s post-hoc test) was used for multiple comparisons. In item (C), an independent samples t-test was applied, and in item (D), a Pearson correlation analysis was conducted. For all in vitro experiments, at least three independent biological replicates were performed (*n* = 3 per group), and data were presented as mean ± standard deviation (SD). ** indicates *P* < 0.01, *** indicates *P* < 0.001, ns indicates no statistical significance

There were complementary binding sites between FBXL19-AS1 and miR-650 (Fig. 3A). Co-transfection experiments using either a miR-650 mimic or inhibitor demonstrated that luciferase activity was significantly reduced or enhanced, respectively, whereas no significant change was observed in the MUT group. These results indicated that miR-650 can directly bind to the wild-type sequence of FBXL19-AS1 (Fig. 3B). miR-650 exhibited a downregulated expression pattern during the pathological progression of AD (Fig. 3C), and its expression was significantly and negatively correlated with that of FBXL19-AS1 (Fig. 3D). In cellular experiments, Aβ25–35 treatment significantly decreased miR-650 expression in SH-SY5Y and BV2 cells. Knockdown of FBXL19-AS1 restored miR-650 expression, and further inhibition of miR-650 reversed this restorative effect (Figs. 3E–F).

### Functional validation of the FBXL19-AS1/miR-650 axis in the Aβ25–35 induced cell model

**Fig. 4:**
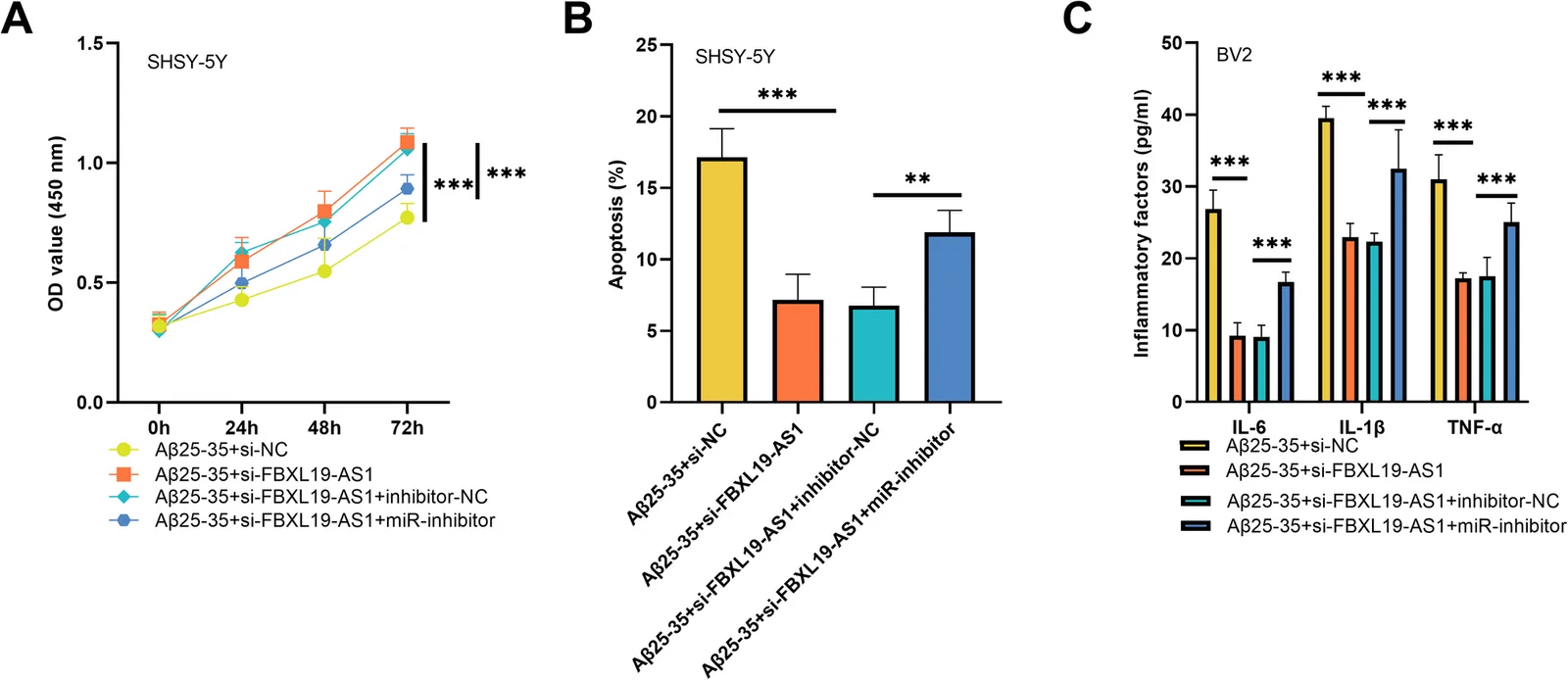
Functional validation of the FBXL19-AS1/miR-650 regulatory axis in an Aβ25–35-induced cellular model. **A**. Knockdown of FBXL19-AS1 promoted SH-SY5Y cell proliferation, while miR-650 inhibition partially reversed this effect. **B**. Knockdown of FBXL19-AS1 suppressed SH-SY5Y cell apoptosis, and miR-650 inhibition partially counteracted this suppression. **C**. Knockdown of FBXL19-AS1 reduced the release of inflammatory cytokines (IL-6, IL-1β, TNF-α) in BV2 cells, and miR-650 inhibition partially reversed this reduction. All data were analyzed using one-way ANOVA followed by Tukey’s post hoc test for multiple group comparisons. For all in vitro experiments, at least three independent biological replicates were performed (*n* = 3 per group), and data were presented as mean ± standard deviation (SD). ** indicates *P* < 0.01, *** indicates *P* < 0.001

Knockdown of FBXL19-AS1 significantly promoted the proliferation of SH-SY5Y cells (Fig. 4A), inhibited cell apoptosis (Fig. 4B), and reduced the release of inflammatory cytokines in BV2 cells (Fig. 4C). However, concurrent inhibition of miR-650 partially reversed the effects induced by FBXL19-AS1 knockdown.

### miR-650 mimic reversed Aβ25-35-induced cell injury and inflammation

To confirm the protective role of miR-650, we transfected SH-SY5Y and BV2 cells with miR-650 mimic before Aβ25–35 exposure. As shown in Supplementary Fig. 2A–D, Aβ25–35 treatment significantly reduced miR-650 expression and increased ITGB3 mRNA levels, while miR-650 mimic effectively reversed both changes. Functional assays revealed that miR-650 mimic significantly restored SH-SY5Y cell viability (Supplementary Fig. 2E) and reduced the apoptosis rate (Supplementary Fig. 2F) compared with the NC mimic group. In BV2 cells, miR-650 mimic markedly decreased the secretion of pro-inflammatory cytokines IL-6, IL-1β, and TNF-α (Supplementary Fig. 2G). These results indicated that miR-650 itself protects against Aβ25-35-induced neuronal injury and neuroinflammation.

### Prediction and functional enrichment analysis of miR-650 target genes

**Fig. 5:**
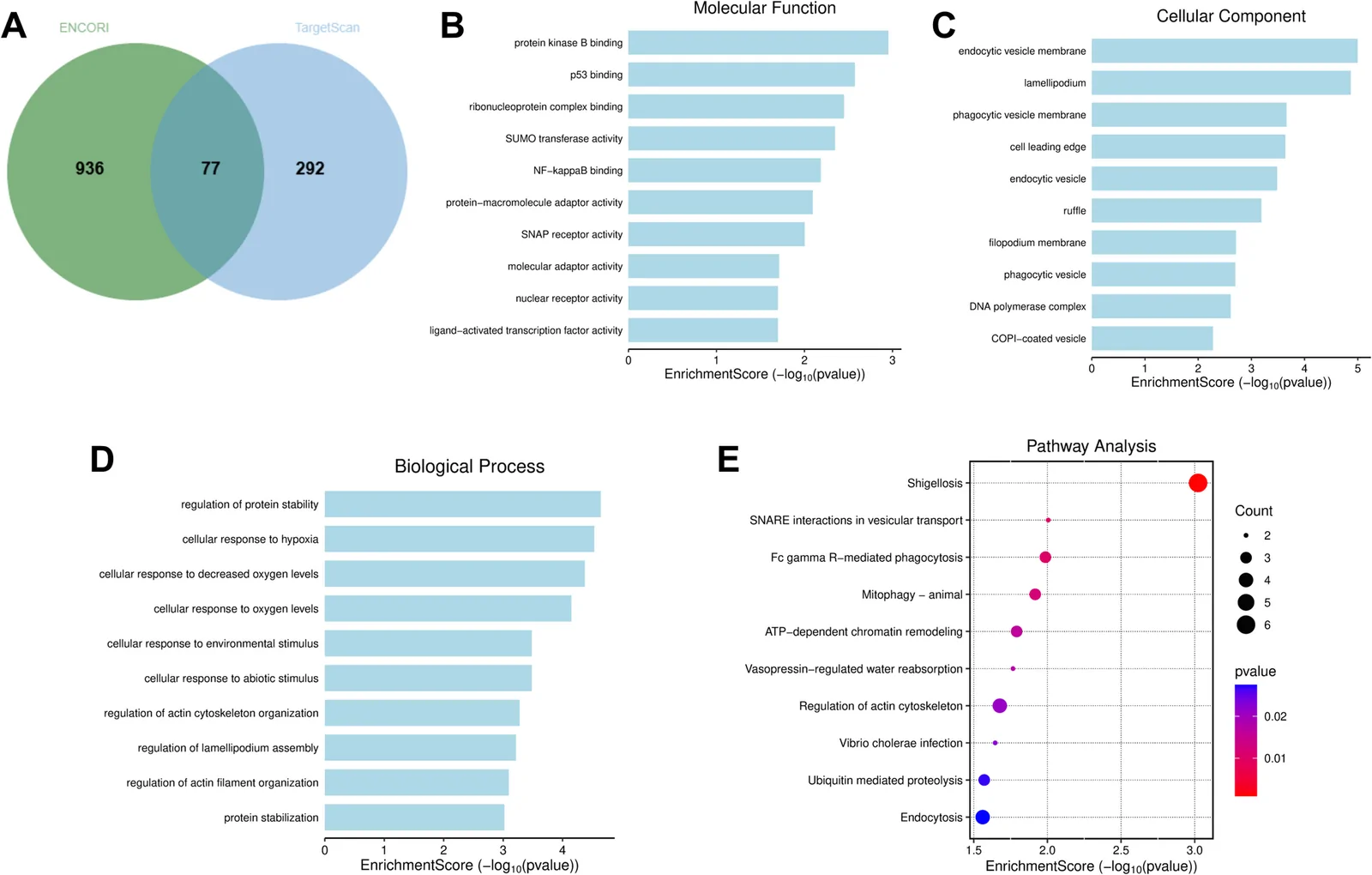
Prediction and functional enrichment analysis of miR-650 target genes. **A**. Venn diagram analysis of miR-650 target genes predicted by ENCORI and TargetScan. **B**. Enrichment analysis of molecular functions for miR-650 target genes. **C**. Enrichment analysis of cellular components for miR-650 target genes. **D**. Enrichment analysis of biological processes for miR-650 target genes. **E**. Pathway enrichment analysis of miR-650 target genes

To explore the potential biological functions of miR-650 in AD, we predicted its target genes using the ENCORI and TargetScan databases. A total of 77 high-confidence overlapping target genes were identified (Fig. 5A). GO enrichment analysis revealed that these target genes were significantly enriched in multiple AD-related functional terms. In the category of Molecular Function, the enriched terms included protein kinase B binding, p53 binding, and nuclear factor kappa-B (NF-κB) binding (Fig. 5B). Cellular Component analysis demonstrated significant enrichment in endocytic vesicle membrane, lamellipodium, and phagocytic vesicle membrane (Fig. 5C). Biological Process analysis uncovered prominent enrichments in the regulation of protein stability, cellular response to hypoxia, regulation of actin cytoskeleton organization, and protein stabilization (Fig. 5D). KEGG pathway analysis indicated that the target genes of miR-650 were significantly enriched in pathways closely associated with AD pathology, including mitophagy, endocytosis, ubiquitin-mediated proteolysis, and regulation of the actin cytoskeleton (Fig. 5E).

#### Targeting the regulatory relationship between miR-650 and ITGB3

**Fig. 6:**
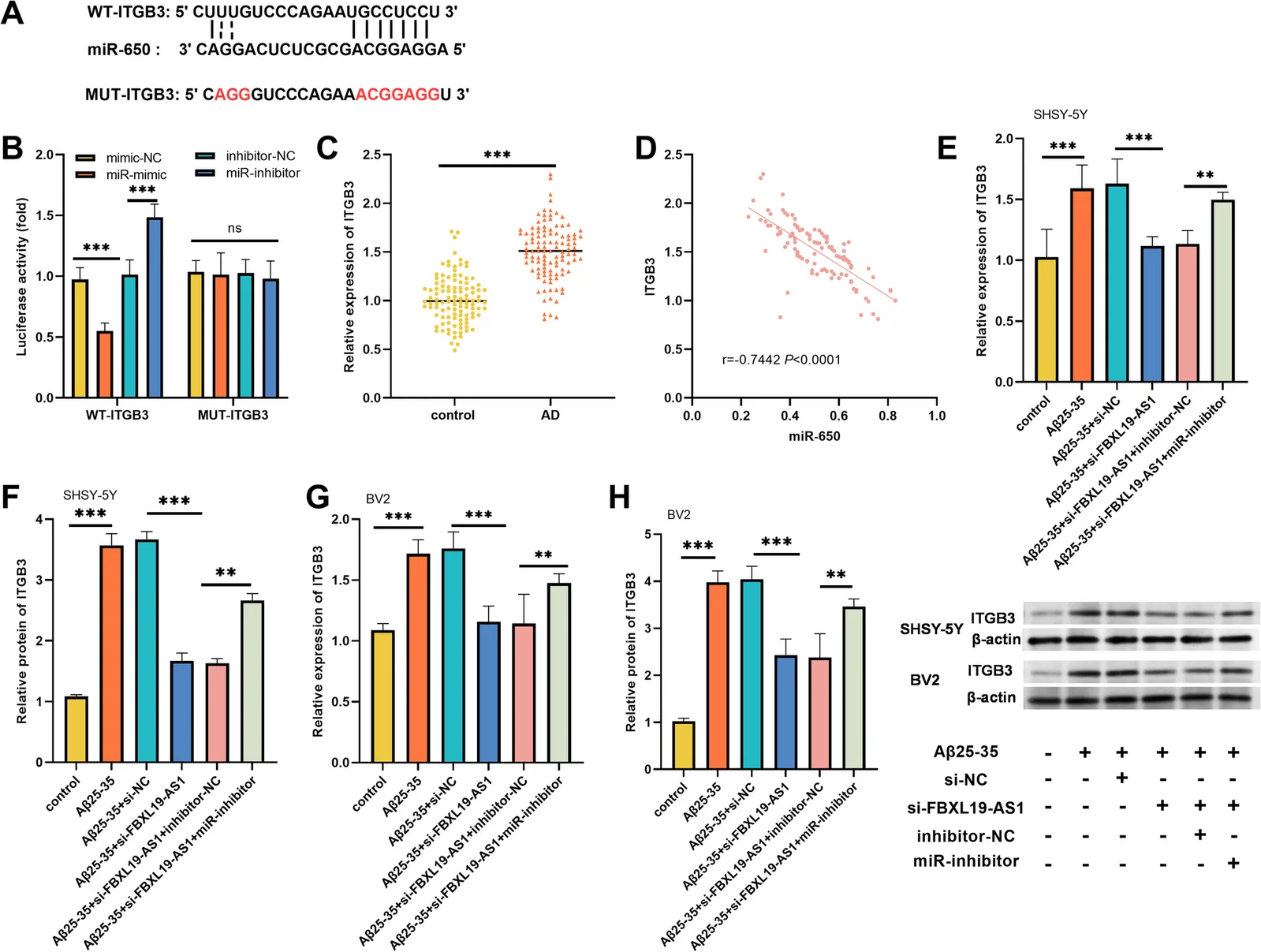
The targeting regulatory relationship between miR-650 and ITGB3. **A**. Complementary sequences between miR-650 and ITGB3. **B**. Luciferase reporter assays confirmed the direct interaction between miR-650 and ITGB3. **C**. ITGB3 may be upregulated during AD pathogenesis. **D**. miR-650 expression was inversely correlated with ITGB3 expression. **E–H**. In Aβ25–35-treated SH-SY5Y (**E-F**) and BV2 (**G-H**) cells, ITGB3 mRNA and protein expression was assessed after modulation of miR-650 or FBXL19-AS1. For items (B, E, F, G, H), a single-factor analysis of variance (with Tukey’s post-hoc test) was used for multiple comparisons. In item (C), an independent samples t-test was applied, and in item (D), a Pearson correlation analysis was conducted. For all in vitro experiments, at least three independent biological replicates were performed (*n* = 3 per group), and data were presented as mean ± standard deviation (SD). ** indicates *P* < 0.01, *** indicates *P* < 0.001, ns indicates no statistical significance

The 3’UTR region of ITGB3 contains binding sites complementary to miR-650 (Fig. 6A). Following transfection with the miR-650 mimic, luciferase activity in the WT group was significantly reduced. Conversely, transfection with the miR-650 inhibitor led to a significant increase in luciferase activity in the WT group. In contrast, no notable changes in luciferase activity were observed in the MUT group under the same experimental conditions, suggesting that miR-650 directly bound to the WT-ITGB3 (Fig. 6B). Furthermore, ITGB3 expression appeared to be upregulated under pathological conditions associated with AD (Fig. 6C), and miR-650 specifically targeted and suppressed ITGB3 expression (Fig. 6D). In both SH-SY5Y (Fig. 6E-F) and BV2 (Fig. 6G-H) cells treated with Aβ25–35, ITGB3 mRNA and protein expression were significantly elevated following Aβ25–35 exposure. Knockdown of FBXL19-AS1 markedly reduced ITGB3 mRNA and protein expression, whereas concurrent inhibition of miR-650 partially reversed this downregulatory effect.

### ITGB3 overexpression abolished the protective effect of FBXL19-AS1 knockdown

To test whether ITGB3 is the key downstream effector of the FBXL19-AS1/miR-650 axis, we performed rescue experiments by co-transfecting si-FBXL19-AS1 with an ITGB3 overexpression plasmid. As shown in Supplementary Fig. 3A–B, si-FBXL19-AS1 significantly reduced ITGB3 mRNA levels, whereas co-transfection with ITGB3 overexpression plasmid restored ITGB3 expression (*P* < 0.01 vs. si-FBXL19-AS1 + empty vector). Functional assays demonstrated that si-FBXL19-AS1 increased SH-SY5Y cell viability (Supplementary Fig. 3C) and decreased apoptosis (Supplementary Fig. 3D), and these protective effects were largely abolished by ITGB3 overexpression. Similarly, in BV2 cells, si-FBXL19-AS1 reduced the secretion of IL-6, IL-1β, and TNF-α, and ITGB3 overexpression reversed this anti-inflammatory effect (Supplementary Fig. 3E). These findings confirmed that FBXL19-AS1 exerted its pro‑AD effects primarily through upregulating ITGB3 via sponging miR-650.

### Triple rescue experiment confirmed the FBXL19-AS1/miR-650/ITGB3 regulatory axis

To further validate the complete signaling cascade, we conducted a triple rescue experiment in Aβ25‑35‑treated SH‑SY5Y and BV2 cells by co‑transfecting si‑FBXL19‑AS1, miR‑650 inhibitor, and ITGB3 overexpression plasmid (Supplementary Fig. 4A–B). As expected, FBXL19‑AS1 knockdown significantly reduced ITGB3 expression, which was partially restored by miR‑650 inhibition. Notably, additional ITGB3 overexpression further rescued ITGB3 levels even in the presence of miR‑650 inhibitor (Supplementary Fig. 4A–B). Functionally, si‑FBXL19‑AS1 improved SH‑SY5Y cell viability and reduced apoptosis, and these protective effects were attenuated by miR‑650 inhibitor; subsequent ITGB3 overexpression again weakened the protection (Supplementary Fig. 4C–D). In BV2 microglial cells, si‑FBXL19‑AS1 decreased the secretion of IL‑6, IL‑1β, and TNF‑α, whereas miR‑650 inhibitor partially restored cytokine levels, and ITGB3 overexpression further reversed the anti‑inflammatory effect (Supplementary Fig. 4E). These results firmly establish that FBXL19‑AS1 promotes AD‑related pathological progression through the miR‑650/ITGB3 axis.

## Discussion

AD has complex pathological mechanisms [25]. Bioinformatics analyses suggest that it may also play a role in the pathogenesis of AD [19]. However, the specific regulatory mechanism of FBXL19-AS1 in AD remained unclear. In this study, we enrolled 110 patients with AD and 110 healthy controls, and established an in vitro AD cellular model by treating SH-SY5Y and BV2 cells with Aβ25–35. For the first time, we systematically investigated the role and underlying mechanisms of the FBXL19-AS1/miR-650/ITGB3 signaling axis in the progression of AD pathology.

Emerging evidence suggests that FBXL19-AS1 may be involved in the modulation of pathways associated with AD [19]. In this study, we observed that FBXL19-AS1 was significantly upregulated in AD patients and exhibited favorable diagnostic performance. Its expression was significantly and negatively correlated with the MoCA score, indicating a potential direct involvement in cognitive impairment. In vitro experiments revealed that Aβ25–35 treatment induced apoptosis in SH-SY5Y cells and triggered inflammatory responses in BV2 cells, accompanied by increased FBXL19-AS1 expression. Notably, knockdown of FBXL19-AS1 partially reversed these pathological changes, suggesting that FBXL19-AS1 may actively contribute to the pathogenesis of AD.

miR-650 is downregulated in rheumatoid arthritis synovial fibroblasts, and its overexpression can significantly inhibit cell proliferation and the release of inflammatory cytokines [26]. Moreover, ITGB3 has been shown to promote the development and progression of pancreatic ductal adenocarcinoma (PDAC) [27]. This study further elucidated its potential mechanism: FBXL19-AS1 functions as a competing endogenous RNA (ceRNA) by sponging miR-650, thereby modulating its activity. The study reported that miR-650 expression was significantly reduced in AD patients. Further experiments demonstrated that miR-650 directly targeted ITGB3, which was highly expressed in AD patients. Consequently, FBXL19-AS1 may promote neuroinflammatory responses and cell apoptosis by suppressing miR-650 expression, thereby alleviating its translational inhibition of ITGB3. This mechanism aligned with previous findings indicating that miR-650 expression in the brains of AD patients was markedly altered, and its overexpression can significantly regulate cyclin-dependent kinase 5 (CDK5) levels, thereby ameliorating AD-related pathological changes [20]. Additionally, ITGB3 has also been implicated in astrocyte reactivity associated with AD [22]. Furthermore, our rescue experiments demonstrated that miR-650 mimic alone was sufficient to reverse Aβ25–35‑induced neuronal injury and neuroinflammation, and that ITGB3 overexpression abolished the protective effects of FBXL19-AS1 knockdown. These results, together with the Western blot data, provide strong evidence that FBXL19-AS1 acts as a ceRNA to sponge miR-650, thereby derepressing ITGB3 expression and promoting AD pathogenesis.

To further explore the broader biological impacts induced by the dysregulation of miR-650 in AD, we performed bioinformatics analysis on its target genes. The analysis results strongly demonstrated that miR-650 was involved in multiple key pathological processes of AD. Specifically, the enrichment results in “regulation of protein stability” and “protein stabilization” were consistent with the well-established role of impaired protein homeostasis in AD, during which the abnormal aggregation of Aβ and hyperphosphorylated tau protein overwhelms the functions of the ubiquitin-proteasome and autophagic clearance systems [28]. Notably, the significant enrichment of the “mitophagy” pathway was particularly noteworthy, as mitochondrial dysfunction and mitophagic defects are increasingly recognized as early and critical events in the pathogenesis of AD [29], leading to oxidative stress and energy exhaustion. In addition, the enrichment of the “endocytosis” pathway and the localization of target genes in endocytic and phagocytic vesicles (e.g., endocytic vesicle membrane, phagocytic vesicle) are consistent with the key role of the endocytic pathway in amyloid precursor protein (APP) processing and Aβ production, as well as the phagocytosis of Aβ aggregates by microglia [30]. The enrichment of “cellular response to hypoxia” and “p53 binding” further links miR-650 to the microenvironment of AD, where chronic hypoperfusion and p53-mediated neuronal apoptosis are common features [31, 32]. Finally, the regulation of actin cytoskeleton organization is crucial for synaptic structure and plasticity, and its disruption may be a marker of early cognitive decline in AD. Therefore, in addition to the direct regulation of ITGB3, miR-650 may also exert pleiotropic effects on AD pathological processes by coordinately regulating the gene networks involved in these interconnected pathways.

Combined with our bioinformatics analyses and existing literature, the FBXL19-AS1/miR-650/ITGB3 axis may be correlated with classic AD pathologies. Given that miR-650 target genes are enriched in endocytosis, phagocytosis, and actin cytoskeleton pathways, increased ITGB3 might impair microglial phagocytosis of Aβ and facilitate amyloid accumulation. Upregulated ITGB3 could also disrupt actin cytoskeleton dynamics and compromise synaptic structure and plasticity. Additionally, the enrichment of p53 and mitophagy pathways implies that this axis potentially induces tau hyperphosphorylation and neuronal apoptosis via disturbing mitochondrial homeostasis. ITGB3 may maintain the pro-inflammatory phenotype of microglia and mediate crosstalk among neuroinflammation, impaired Aβ clearance, and mitochondrial dysfunction. Further investigations are required to verify these hypotheses in future studies.

It is important to consider whether the expression patterns of FBXL19-AS1, miR-650, and ITGB3 in peripheral blood reflect their status within the central nervous system (CNS) in AD. Accumulating evidence suggests that circulating lncRNAs and miRNAs can serve as surrogate biomarkers for CNS pathologies, as they are released from brain cells into the bloodstream via exosomes or other mechanisms and exhibit stable expression that correlates with brain tissue levels [33–35]. Specifically, a recent study demonstrated that FBXL19-AS1 is among the differentially expressed lncRNAs identified in AD brain tissues by RNA sequencing [19]. Moreover, miR-650 has been shown to be downregulated in AD brains, where its overexpression ameliorates AD-related pathology through targeting CDK5 [20]. ITGB3 upregulation has also been observed in astrocytes of AD mouse brains [22]. Although direct comparison between paired peripheral blood and brain tissue from the same individuals is not feasible in living patients, the consistency of expression directions (upregulation of FBXL19-AS1 and ITGB3, downregulation of miR-650) across peripheral blood in our study and previously reported brain tissue data supports the use of peripheral blood as a practical proxy for investigating this regulatory axis in AD.

Clinically, FBXL19-AS1 may serve as a supplementary biomarker for the early diagnosis and assessment of AD. In routine physical examinations of middle-aged and elderly individuals, the combined measurement of FBXL19-AS1 levels in peripheral blood with traditional cognitive assessments may enhance the early detection rate of AD, particularly in patients presenting with atypical clinical symptoms. Continuous monitoring of FBXL19-AS1 expression dynamics may provide a basis for timely adjustments in intervention strategies.

This study investigated the molecular mechanisms of Alzheimer’s disease (AD) solely based on two immortalized cell lines, SH-SY5Y neuronal cells and BV2 microglia, which inherently imposes multiple limitations on our conclusions. For one thing, we failed to adopt primary mouse cortical neurons and astrocytes for validation; primary neuronal cells retain more authentic native neuronal phenotypes and possess superior physiological consistency with brain tissues in vivo, so evidence from primary cells is still absent in our current work. For another, all experimental verifications were restricted to the cellular level, without in vivo functional validation using AD animal models. Beyond the cell experimental deficiencies, there are notable drawbacks in our clinical sample design. All clinical specimens were collected merely from the Affiliated Hospital of Zunyi Medical University, and single-center sampling is likely to cause regional and hospital-specific biases, reducing the extrapolation capacity of our research results. Besides, our clinical cohort only contained 110 AD patients and 110 healthy controls, representing a limited sample scale; larger multicenter cohorts are required to further confirm the diagnostic value of FBXL19-AS1 and enable stratified subgroup analyses according to AD pathological stages or different APOE genotypes. Furthermore, we have not acquired direct experimental proof connecting the FBXL19-AS1/miR-650/ITGB3 regulatory axis to the core pathological markers of AD, namely Aβ metabolism and tau hyperphosphorylation. Even though GO and KEGG enrichment analyses offered tentative mechanistic hints: FBXL19-AS1/miR-650-mediated ITGB3 expression may modulate microglial Aβ clearance through endocytosis, and enrichment of mitophagy and p53 binding pathways indicated potential crosstalk between this molecular axis and tau-triggered cell cycle re-entry as well as mitochondrial dysfunction, we did not quantitatively detect the changes in Aβ40/Aβ42 and phosphorylated tau protein levels after overexpressing or silencing this regulatory axis in current cell experiments. Additionally, whether FBXL19-AS1 exerts other pathogenic effects through distinct downstream target genes still remains unexplored. To compensate for all the above shortcomings, we have formulated corresponding follow-up research schemes. In future studies, we will enroll clinical samples from multiple geographically diverse medical institutions to expand the sample size and eliminate single-center bias. We will also establish AD animal models to validate the biological function of the FBXL19-AS1/miR-650/ITGB3 axis in vivo, and conduct biochemical quantification of Aβ and phosphorylated tau proteins to establish a definite causal relationship between this molecular cascade and central AD pathological processes. Meanwhile, primary cortical neuron cultures will be introduced to supplement more physiologically relevant cellular evidence, and we will further explore other downstream target genes of FBXL19-AS1 to fully dissect its complete pathogenic regulatory network in AD.

## Conclusion

In summary, the findings of this study suggest that FBXL19-AS1 may interact with and modulate miR-650, thereby affecting the expression of ITGB3 and potentially contributing to the pathological progression of AD. However, these conclusions should be interpreted with caution, given the study’s limitations, including the single-center design, relatively modest sample size, and lack of in vivo animal validation. Further investigations are warranted to confirm the role of the FBXL19-AS1/miR-650/ITGB3 axis in AD.

## Supplementary Information

Supplementary Material 1. Subcellular localization of FBXL19-AS1 in SH-SY5Y and BV2 cells. **A-B**. Nuclear and cytoplasmic fractions were separated, and the distribution of FBXL19-AS1 was analyzed by RT-qPCR. U6 and GAPDH mRNA were used as nuclear and cytoplasmic markers, respectively. For all in vitro experiments, at least three independent biological replicates were performed (*n* = 3 per group), and data were presented as mean ± standard deviation (SD).

Supplementary Material 2. miR-650 mimic reversed Aβ25-35-induced cell injury and inflammation. **A–D**. RT-qPCR analysis of miR-650 (A-B) and ITGB3 mRNA (C-D) in SH-SY5Y cells and BV2 cells. **E**. CCK-8 assay showing SH-SY5Y cell viability. **F**. Flow cytometry analysis of SH-SY5Y apoptosis. **G**. ELISA quantification of IL-6, IL-1β, and TNF-α in BV2 cell supernatant. All data were analyzed using one-way ANOVA followed by Tukey’s post hoc test for multiple group comparisons. For all in vitro experiments, at least three independent biological replicates were performed (*n* = 3 per group), and data were presented as mean ± standard deviation (SD). *** indicates *P* < 0.001.

Supplementary Material 3. ITGB3 overexpression abolished the protective effect of FBXL19-AS1 knockdown. **A–B**. RT-qPCR analysis of ITGB3 mRNA in SH-SY5Y (A) and BV2 (B) cells after co-transfection with si-FBXL19-AS1 and ITGB3 overexpression plasmid (or empty vector). **C**. CCK-8 assay showing SH-SY5Y cell viability. **D**. Flow cytometry analysis of SH-SY5Y apoptosis. **E**. ELISA quantification of IL-6, IL-1β, and TNF-α in BV2 cell supernatant. All data were analyzed using one-way ANOVA followed by Tukey’s post hoc test for multiple group comparisons. For all in vitro experiments, at least three independent biological replicates were performed (*n* = 3 per group), and data were presented as mean ± standard deviation (SD). *** indicates *P* < 0.001.

Supplementary Material 4. Triple rescue experiment validating the FBXL19-AS1/miR-650/ITGB3 axis in Aβ25‑35‑treated SH‑SY5Y and BV2 cells. **A–B**. Relative ITGB3 expression in SH‑SY5Y (A) and BV2 (B) cells after co‑transfection with si‑FBXL19‑AS1, miR‑650 inhibitor, and ITGB3 overexpression plasmid. **C**. CCK‑8 assay showing SH‑SY5Y cell viability. **D**. Flow cytometry analysis of SH‑SY5Y apoptosis. **E**. ELISA quantification of IL‑6, IL‑1β, and TNF‑α in BV2 cell supernatant. FBXL19‑AS1 knockdown reduced ITGB3 expression, improved cell viability, decreased apoptosis, and lowered inflammatory factors. miR‑650 inhibition partially reversed these effects, and additional ITGB3 overexpression further counteracted the protective phenotype. Data were analyzed using one‑way ANOVA with Tukey’s post hoc test. All experiments were performed with at least three independent biological replicates (*n* = 3 per group), and data are presented as mean ± SD. ** indicates *P* < 0.01. *** indicates *P* < 0.001.

Supplementary Material 5. Public database validation using AlzData. ITGB3 expression in the hippocampus of AD patients vs. controls.

## Data Availability

Data available on request from the authors.
